# Modified Antibiotic Adjuvant Ratios Can Slow and Steer the Evolution of Resistance: Co-amoxiclav as a Case Study

**DOI:** 10.1128/mBio.01831-19

**Published:** 2019-09-17

**Authors:** Richard C. Allen, Sam P. Brown

**Affiliations:** a Institute of Integrative Biology, ETH Zürich, Zürich, Switzerland; b School of Biological Sciences, Georgia Institute of Technology, Atlanta, Georgia, USA; c Center for Microbial Dynamics and Infection, Georgia Institute of Technology, Atlanta, Georgia, USA; Emory University

**Keywords:** antimicrobial chemotherapy, adjuvants, antibiotic resistance, antimicrobial combinations, beta-lactamases, evolution, plasmid-mediated resistance

## Abstract

As antibiotic resistance spreads, developing sustainable methods to restore the efficacy of existing antibiotics is increasingly important. One widespread method is to combine antibiotics with synergistically acting adjuvants that inhibit resistance mechanisms, allowing drug killing. Here we use co-amoxiclav (a clinically important combination of the β-lactam antibiotic amoxicillin and the β-lactamase inhibitor clavulanate) to ask whether treatment efficacy and resistance evolution can be decoupled via component dosing modifications. A simple mathematical model predicts that different ratios of these two drug components can produce distinct evolutionary responses irrespective of the initial efficacy. We test this hypothesis by selecting Escherichia coli with a plasmid-encoded β-lactamase (CTX-M-14), against different concentrations of amoxicillin and clavulanate. Consistent with our theory, we found that while resistance evolved under all conditions, the component ratio influenced both the rate and mechanism of resistance evolution. Specifically, we found that the current clinical practice of high amoxicillin-to-clavulanate ratios resulted in the most rapid adaptation to antibiotics via gene dosing responses. Increased plasmid copy number allowed E. coli to increase β-lactamase dosing and effectively titrate out low quantities of clavulanate, restoring amoxicillin resistance. In contrast, high clavulanate ratios were more robust—plasmid copy number did not increase, although porin or efflux resistance mechanisms were found, as for all drug ratios. Our results indicate that by changing the ratio of adjuvant to antibiotic we can slow and steer the path of resistance evolution. We therefore suggest using increased adjuvant dosing regimens to slow the rate of resistance evolution.

## Abstract (precis)

As antibiotic resistance spreads, a promising approach is to restore the effectiveness of existing drugs via coadministration with adjuvants that inhibit resistance. However, as for monotherapy, antibiotic-adjuvant therapies can select for a variety of resistance mechanisms, so it is imperative that adjuvants be used in a sustainable manner. We test whether the rate of resistance evolution can be decoupled from treatment efficacy using co-amoxiclav, a clinically important combination of the β-lactam amoxicillin and β-lactamase inhibitor clavulanate. Using experimental evolution and a simple theoretical model, we show that the current co-amoxiclav formulation with a high proportion of amoxicillin rapidly selects for resistance via increased β-lactamase production. On the other hand, formulations with more clavulanate and less amoxicillin have similar efficacies yet prevent the selective benefit of increased β-lactamase. We suggest that by blocking common paths to resistance, treatment combinations with the adjuvant in excess can slow the evolution of resistance.

## INTRODUCTION

The current crisis of antibiotic resistance is grounded in the ability of bacterial pathogens to rapidly evolve and adapt to novel stressors like antibiotics (1). Even for the same drug, many different mechanisms confer resistance, often with distinct transmissibility, costs, and cross-resistance (2–7). Examples of resistance have been found for all currently used antibiotics, and recently, clinicians have begun to face pathogens that are resistant to all available antibiotics (4, 8–10).

In addition to the ongoing search for new drugs (11), an important direction in combating resistance is the restoration of antibiotic sensitivity to existing drugs via the use of antiresistance compounds or adjuvants (12, 13). Antibiotic adjuvants are compounds that have little or no effect on bacterial growth in isolation but instead enhance the activity of antibiotics (14). Adjuvants reduce the efficacy of active or passive mechanisms of bacterial resistance; for example, they can inhibit efflux pumps (15), increase membrane permeability (16), disrupt biofilm formation (17), or inhibit enzymes that degrade or modify antibiotics (18).

β-Lactamase inhibitors represent the best understood adjuvants (12, 14), representing important compounds in clinical use (19) and important new potential drugs (20). β-Lactamase inhibitors mimic β-lactam antibiotics (and can inhibit growth at high concentrations), but at low concentrations, they inhibit β-lactamase enzymes, preventing them from degrading β-lactam antibiotics (21). β-Lactamase-mediated resistance is especially problematic for Gram-negative pathogens where these enzymes are common and disseminated on plasmids (22). In this context, β-lactam plus β-lactamase inhibitor (BLBLI) combinations are a critical therapeutic strategy, restoring β-lactam sensitivity without using antibiotics of last resort like carbapenems (23).

In this study, we use co-amoxiclav (brand name Augmentin), a BLBLI combination of amoxicillin and clavulanate (clavulanic acid) that has been used globally since 1981 (24), and is on the WHO list of essential medicines (19). Amoxicillin is a bactericidal β-lactam antibiotic that inhibits synthesis of the bacterial cell wall. The structure of the adjuvant clavulanate is similar to the structures of β-lactam antibiotics, and therefore, it acts as a competitive inhibitor of many β-lactamase enzymes (21). By preventing amoxicillin cleavage, clavulanate suppresses the resistance phenotype, making amoxicillin effective against strains that would be resistant in the absence of clavulanate.

Despite the efficacy of BLBLI combinations like co-amoxiclav, resistance is still possible either by altering the effect of amoxicillin or the effect of clavulanate. Clavulanate is ineffective against resistance mechanisms that do not involve β-lactamase expression. Thus, direct resistance to amoxicillin via altered penicillin binding protein structure, reduced porin expression, or increased efflux pump expression can lead to resistance to co-amoxiclav (21). On the other hand, increased production of β-lactamase enzymes can overwhelm the clavulanate (25), and inhibitor-resistant β-lactamase enzymes can reduce (or abolish) the effect of clavulanate (26).

Despite the recent interest in adjuvants, the relative doses of the components in adjuvant therapies have received little attention, with clinical amoxicillin/clavulanate ratios varying from 2:1 to 16:1 (27), with an increase in amoxicillin more recently to combat resistance (24). Here we mathematically model and empirically map the synergy between amoxicillin and clavulanate in inhibiting β-lactamase-expressing Escherichia coli. We then go on to experimentally validate model predictions that drug ratios that have similar initial levels of control (measured by effect on final bacterial density) can produce distinct evolutionary responses. Specifically, we find that current high amoxicillin ratios lead to the rapid evolution of resistance via increased β-lactamase expression, while low amoxicillin ratios with similar initial efficacies are more robust and maintain the efficacy of our meager pool of β-lactamase inhibitors. We therefore suggest the use of increased clavulanate dosing regimens to slow the rate of resistance evolution.

## RESULTS

### Theoretical model.

We begin by developing a simple qualitative model (equation 1) for the control efficacy *A* (proportional reduction in bacterial density at 22 h compared to control) of co-amoxiclav. First, to consider the action of β-lactam antibiotic (dose *a*) on a drug-sensitive target, we assume diminishing returns so that *A = a^x^* (where *x *<* *1). Next, we introduce a β-lactamase-mediated resistance mechanism with efficacy *r*, so that *A = a^x^*(1 − *r*). The introduction of β-lactamase-inhibiting adjuvant (dose *b*) can now be incorporated: *A = a^x^*[1 − *r*(1 − *b^y^*)], where *y *<* *1 captures diminishing returns on the adjuvant. Finally, we can incorporate a β-lactamase-independent mechanism of resistance (e.g., porins or efflux) with efficacy *s*:

(1) $$A=a^{x}\left[1-r\left(1-b^{y}\right)\right](1-s)$$

**FIG 1:**
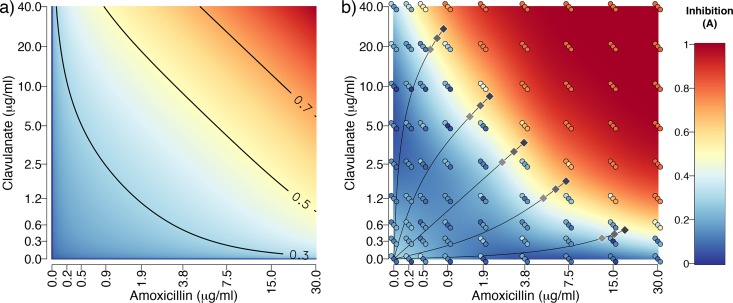
Synergistic effects of amoxicillin and clavulanate when inhibiting growth of a β-lactamase-expressing bacterium. (a) Predicted inhibition (*A*) from a theoretical model of control of a β-lactamase-expressing bacterium (equation 1). The color gradient shows values as highlighted by the contours. The model is illustrated using parameters fitted to the data in panel b: *a =* amoxillicin/amoxicillin_max (30 μg/ml), *b =* clavulanate/clavulanate_max (40 μg/ml); *x = y = *0.198, *r *=* *0.952. (b) Measured inhibition of ancestral E. coli expressing β-lactamase from a plasmid. The circles represent three replicate measures, while the surface is a loess smoothed fit. For ease of comparison with the theoretical model, inhibition was calculated from growth after 22 h by subtracting growth from the maximum measured growth value (OD_600_ of 0.81). The gray diamonds show the 15 selection conditions used for the experimental evolution, with the darkest diamonds representing stronger inhibition. Note that in both plots, the axes have been transformed (inverse hyperbolic sine) to show the data more clearly.

The model predicts that the two drug components *a* and *b* will show synergy when controlling a pathogen with an existing β-lactamase gene (*r * > * *0), as increasing one component increases the marginal value of the other [the term *dA*^2^/(*da db*) is positive; Fig. 1a]. This prediction is supported by our checkerboard assay experimental data (Fig. 1b). To assess synergy, we first note that neither amoxicillin alone nor clavulanate alone has significant effects on growth of the ancestor over the measured concentrations (effect of amoxicillin alone *F*_1,25_ = 0.88, *P* > 0.1; effect of clavulanate alone *F*_1,25_ = 1.43, *P* > 0.1). Under the assumption of Bliss independence (28, 29), we would therefore expect no reduction when the drugs are combined at these concentrations. In contrast, we observe strong inhibition in the top right portion of Fig. 1b, demonstrating clear synergy between the two components when treating a β-lactamase-expressing target organism. This synergistic effect can be observed in the overview of the inhibition pattern in Fig. 1b, and in the same data when measured with CFU (see Fig. S1 in the supplemental material).

10.1128/mBio.01831-19.1 FIG S1 Checkerboard assay for amoxicillin and clavulanate using colony-forming units (CFU) (assessed by plating) to determine growth. The surface is a loess fit to the data points shown, which are also colored according to the same scale. The color scale has been truncated at the upper limit to better show the pattern in the majority of the data. The black circles show points above the upper limit on the color scale. The diamonds show the concentrations of the components used for the 15 different selective environments. Download FIG S1, TIF file, 1.0 MB. Copyright © 2019 Allen and Brown. 2019 Allen and Brown This content is distributed under the terms of the Creative Commons Attribution 4.0 International license.

Next, we focus on the pathogen and ask how the composition of the drug treatment, described by *a* and *b*, affects the marginal value to the pathogen of increasing β-lactamase resistance (−*dA*/*dr*) or non-β-lactamase resistance (−*dA*/*ds*)? Partially differentiating equation 1, we find:

(2) $$-\frac{\delta A}{\delta s}=a^{x}\left[1-r\left(1-b^{y}\right)\right]$$

(3) $$-\frac{\delta A}{\delta r}=a^{x}\left(1-b^{y}\right)\left(1-s\right)$$

**FIG 2:**
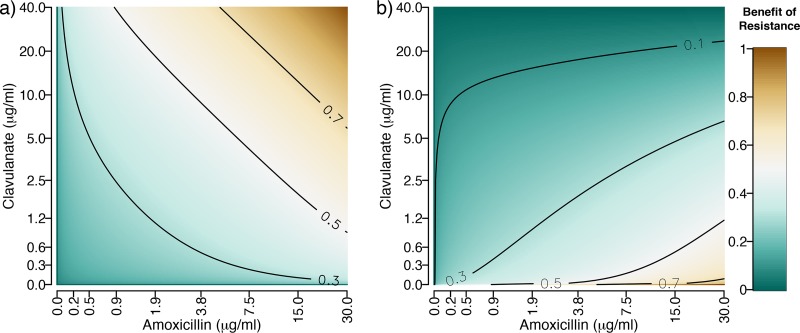
Predicted effect of direct (non-β-lactamase-mediated) mechanisms (−*dA*/*ds*, equation 2) (a) and β-lactamase-mediated mechanisms (−*dA*/*dr*, equation 3) (b) in resisting different doses of amoxicillin and clavulanate. The color gradient and contours show the strength of the selective effect values, with brown showing increased selection for resistance. Note that in both plots, the axes have been transformed (inverse hyperbolic sine) to show the patterns in the predictions more clearly. The parameters are the same as in Fig. 1a.

The model predicts that non-β-lactamase resistance mutations (increases in *s*) will be selected in proportion to the efficacy of the combination treatment (equation 2 and Fig. 2a). In contrast, β-lactamase overproduction mutants (increases in *r*) show an interesting pattern with maximal selection biased toward high amoxicillin ratios (equation 3 and Fig. 2b), as increasing β-lactamase can then effectively titrate out the low concentration of clavulanate and restore the resistance phenotype.

### Adaptation of E. coli to drug environments.

**FIG 3:**
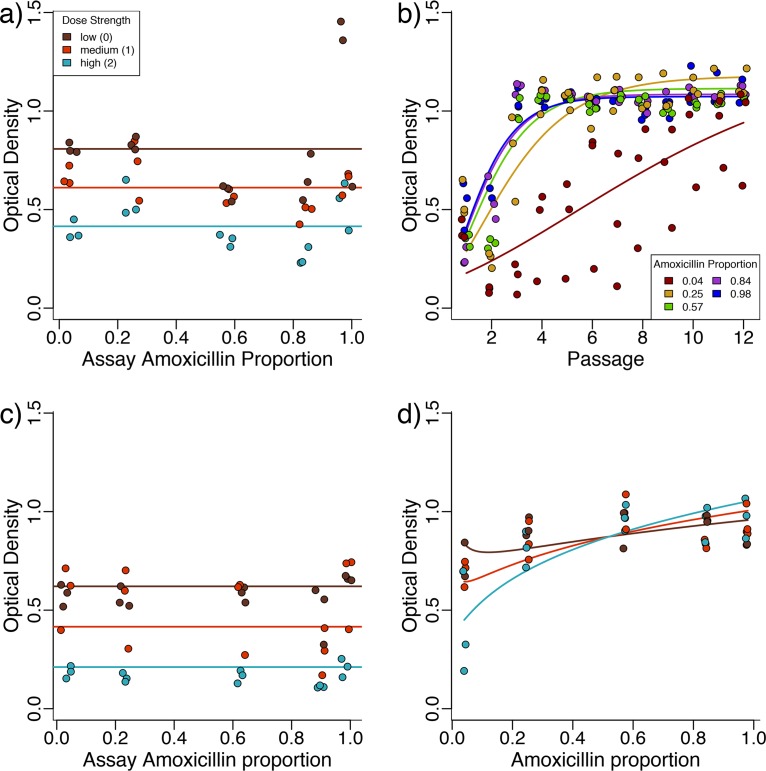
β-Lactamase-expressing E. coli evolves resistance more rapidly to high-amoxicillin/low-clavulanate compositions. (a) The growth of the ancestral strain in different drug environments is affected by the dose strength (shown by color) of the drug combination but is not significantly affected by the amoxicillin proportion of the drug combination. (b) Adaptation of lines selected at high dose strengths of various amoxicillin proportions (shown by color) shows that adaptation to low amoxicillin proportions is slower (the data points for passage 1 are a repeat of the high-dose data for panel a). (c) Control evolution lines are again affected by the dose strength of the drug but are not significantly affected by amoxicillin proportion. (d) The growth of drug-selected lines evolved for six passages in their selection environment is higher for strains evolved at high-amoxicillin/low-clavulanate compositions.

In sum, our model predictions state that amoxicillin-clavulanate treatment compositions with high proportions of amoxicillin will lead to more rapid evolution of resistance via β-lactamase adaptation (Fig. 2b), whereas other resistance mechanisms are not sensitive to changes in composition (Fig. 2a). We next tested our theoretical predictions by conducting experimental evolution in 15 drug environments (shown via diamonds in Fig. 1b and listed in Table 1). The 15 drug environments corresponded to five different amoxicillin ratios (concentration of amoxicillin relative to clavulanate), each at three, independently varying, dose strengths (absolute concentrations of amoxicillin and clavulanate for a given ratio). The five differing ratios were chosen to fall along a treatment efficacy isocline (“high dose”, dark diamonds, Fig. 1b) showing comparable effects on the growth of the ancestor over 22 h. The low- and medium-dose treatments are 70% and 85% concentrations of the high-dose treatments (Table 1). In line with our experimental design, the ancestral strain grew to a lower final density at higher dose strengths (β = −1.96, *F*_1,43_ = 35.3, *P* < 0.001, Fig. 3a) but amoxicillin proportion did not significantly affect the final optical density (*F*_1,43_ = 0.21, *P* > 0.5, Fig. 3a). Measuring growth via CFU (Fig. S1) rather than triplicate optical density (OD) measures (Fig. 1b) shows a very similar synergistic pattern, but with some deviation in the position of the high-dose isocline. As a result of this deviation, CFU measurements indicate a greater inhibition under lower amoxicillin proportions (β = 5967, *F*_1,5_ = 13.9, *P* < 0.05, Fig. S1 and S2). We note that resistance generally evolves faster under stronger inhibition (3, 30), making our experimental design a conservative test of the prediction that resistance evolution will be slower under low-amoxicillin/high-clavulanate treatment regimens.

**TABLE 1:** Concentrations of amoxicillin and clavulanate used for the drug environments used in this paper^*a*^

Amoxicillin proportion	Drug component	Drug concentration (μg/ml) at the following dose strength:		
		Low [0]	Medium [1]	High [2]
0.04	Amoxicillin	0.595	0.723	**0.850**
	Clavulanate	19.0	23.1	**27.2**
0.25	Amoxicillin	1.47	1.79	**2.10**
	Clavulanate	5.89	7.14	**8.40**
0.57	Amoxicillin	2.59	3.15	**3.70**
	Clavulanate	2.59	3.15	**3.70**
0.84	Amoxicillin	5.04	6.12	**7.20**
	Clavulanate	1.26	1.53	**1.80**
0.98	Amoxicillin	12.6	15.3	**18.0**
	Clavulanate	0.394	0.478	**0.523**

a For each of the drug ratios (defined by the amoxicillin proportion), there are three drug doses corresponding to increasing strength of selection. Amoxicillin proportion is calculated as the proportion of amoxicillin in the combination, where both concentrations are relative to their maximum concentrations (maximum amoxicillin concentration [*a*_max_] of 30 and maximum clavulanate concentration [*b*_max_] of 40). The concentrations (in micrograms per milliliter) at the different dose strengths are 70%, 85%, and 100% of the high-dose strength (shown in boldface type). The numbers in brackets (0, 1, and 2) show how dose strength was coded in the statistical models.

10.1128/mBio.01831-19.2 FIG S2 Growth of the ancestral strain after 22 hours of growth in the presence of co-amoxicillin at different amoxicillin proportions (at high doses), measured using CFU counts. Each data point is a single measurement. The blue points represent the five amoxicillin proportions used for selection experiment. The two red points were additional amoxicillin proportions of 0.10 and 0.18 used for some phenotyping. The concentrations of amoxicillin were 1.31 and 1.75 μg/ml, paired with clavulanate concentrations of 15.7 and 10.3 μg/ml. The model is fitted to all points, but omission of the red points does not qualitatively affect conclusions. Download FIG S2, TIF file, 0.02 MB. Copyright © 2019 Allen and Brown. 2019 Allen and Brown This content is distributed under the terms of the Creative Commons Attribution 4.0 International license.

Consistent with our main prediction, we found that adaptation to high-amoxicillin/low-clavulanate treatments resulted in more rapid evolution of resistance (Fig. 3b and Fig. S3, effect of amoxicillin proportion on rate of increase [parameter *k*, methods], β = 0.14, *F*_1,488_ = 274.7, and *P* < 0.001), despite showing similar effects on the yield of the ancestral strain (Fig. 3a). To investigate this more closely, we revived the populations that were selected for six passages (approximately 40 generations) and assayed them for growth in the drug environment they were selected in. While the control strain (evolved in the absence of drugs) grew similarly across amoxicillin proportions (Fig. 3c, main effect of amoxicillin proportion, χ^2^_1_ = 0.66 and *P* > 0.1), the growth of the drug-exposed populations, in their drug environment, was greater for populations selected at high amoxicillin proportions, particularly at high dose strengths (Fig. 3d, dose strength by selective amoxicillin proportion interaction, β = 0.044, χ^2^_1_ = 9.95, and *P* < 0.01). Note that the model in Fig. 3d is the same as that used for the varying dose ratios in the next section, but using a separate model for these data alone gave qualitatively similar results. A similar pattern of growth to Fig. 3d can also be seen in a separate repeat of the experiment where growth was measured using CFU (Fig. S4).

10.1128/mBio.01831-19.3 FIG S3 Strains exposed to high clavulanate drug treatments adapt poorly compared with strains exposed to low clavulanate treatments over the course of selection. Optical density of experimental evolution passages after a 22-hour growth cycle.(a) Drug environments that were used to select the lines shown in panels b, c, and d, which go from low to high treatment doses, with amoxicillin proportion that selection lines were exposed to shown by color. Curves are the fixed effects from a nonlinear mixed-effects model according to the Gompertz equation (see Materials and Methods). Panel d is the same as Fig. 3d in the main article but is repeated here for ease of comparison. Download FIG S3, TIF file, 0.2 MB. Copyright © 2019 Allen and Brown. 2019 Allen and Brown This content is distributed under the terms of the Creative Commons Attribution 4.0 International license.

10.1128/mBio.01831-19.4 FIG S4 Density (measured in CFU) of evolved strains in the after 22 hours of growth in the drug environment (dose strength and amoxicillin proportion) that they were selected in. Each point corresponds to a single measurement from an independent selection line. The fitted lines come from a minimal fixed-effects model on the sqrt of CFU, where dose strength has a negative effect on growth (β = −4824, *F*_1,42_ = 11.23, and *P* < 0.01) and the amoxicillin proportion has a positive effect on growth (β = 5077, *F*_1,42_ = 25.68, and *P* < 0.0001). Download FIG S4, TIF file, 0.1 MB. Copyright © 2019 Allen and Brown. 2019 Allen and Brown This content is distributed under the terms of the Creative Commons Attribution 4.0 International license.

### Cross-resistance between drug environments with different amoxicillin-clavulanate compositions.

**FIG 4:**
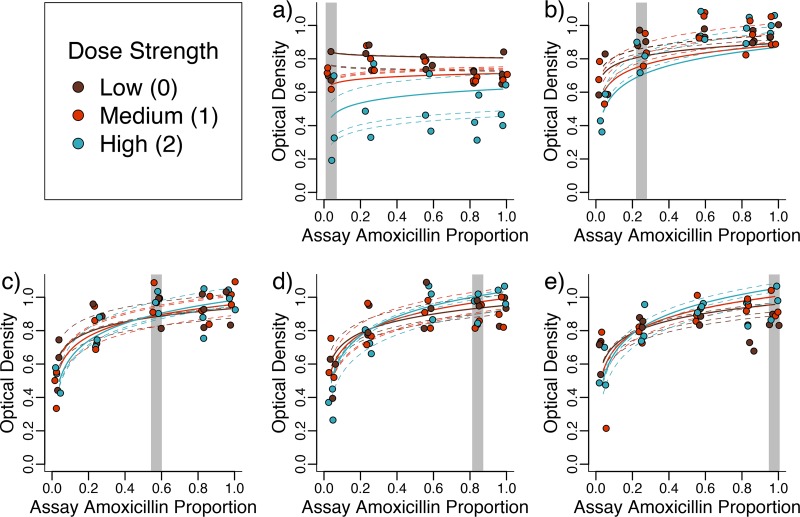
Strains selected under high amoxicillin/low clavulanate adapt to their environment better and are more greatly affected by the amoxicillin proportion of the drug environment. Optical density after 22-h growth is shown for strains adapted to each of five amoxicillin ratios, at different dose strengths, and then assayed at different amoxicillin proportions (but the same dose strength). (a to e) Lines adapted to amoxicillin proportions of 0.04, 0.25, 0.57, 0.84 and 0.98, respectively (highlighted by the gray region). Lines are the fits of a mixed-effects model detailed in the text, with dashed lines showing the fits for the individual evolved lines.

The model suggests that variation in the speed of adaptation at different amoxicillin proportions results from an inability of increased β-lactamase production mutations to confer effective resistance in high-clavulanate environments. We therefore hypothesize that β-lactamase-mediated resistance selected at high amoxicillin proportions is ineffective at low amoxicillin proportions, while the resistance selected at low amoxicillin proportions (direct resistance) is unaffected by amoxicillin proportion. To investigate this, we explored how adaptation to one drug environment influenced growth across distinct drug environments. The populations that had been selected for six passages were exposed to alternate drug environments, first by varying the amoxicillin proportion of the drug environment (while keeping dose strength the same). These data can be seen in Fig. 4.

We found that evolved strains grew better when exposed to high amoxicillin proportions, especially those evolved (and treated) with high dose strengths (assay amoxicillin proportion by dose strength interaction, β = 0.031, χ^2^_1_ = 24.2, and *P* < 0.0001). We found that in addition to growing better when exposed to the dose ratio they had been selected against, lines that were evolved at a high amoxicillin proportion were also affected more by the amoxicillin proportion that they were assayed at (selective amoxicillin proportion by assay amoxicillin proportion interaction, β = 0.037, χ^2^_1_ = 62.5, and *P* < 0.0001).

In summary, we found support for our theoretical predictions: lines selected under high-amoxicillin compositions grew less well in low-amoxicillin/high-clavulanate compositions compared to their performance in high amoxicillin, whereas lines selected in low-amoxicillin compositions grew equally poorly at all amoxicillin ratios.

### Cross-resistance between environments with different dose strengths.

**FIG 5:**
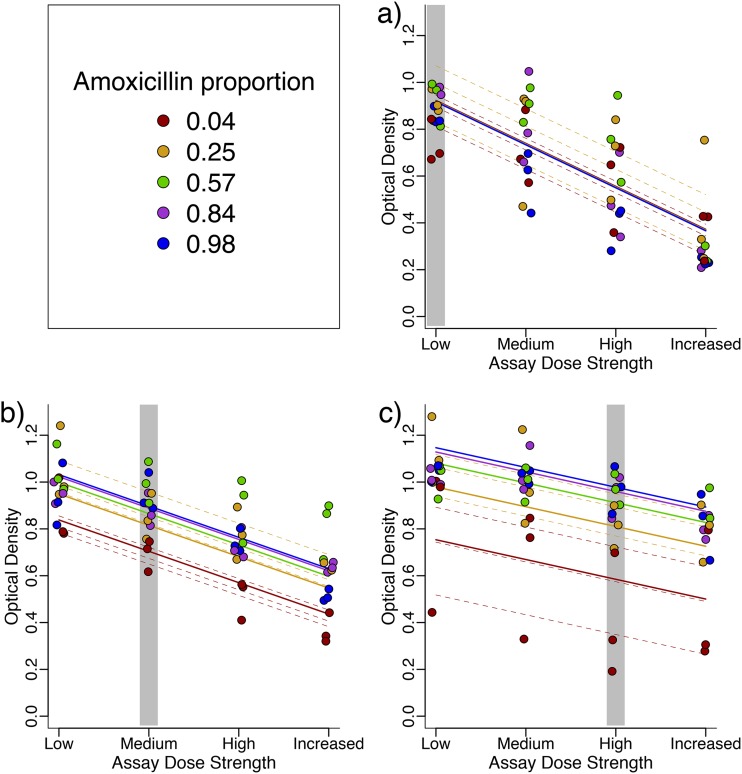
Strains selected at high dose strengths adapt to their environment better and are less strongly affected by increases in the drug dose. Optical density after 22-h growth is shown for strains adapted to (a) low, (b) medium, and (c) high dose strengths and assayed at all dose strengths (but same amoxicillin proportion). Lines are the fits of a mixed-effects model detailed in the text, with dashed lines showing the fits for the individual evolved lines. The gray areas indicate the dose strength the lines were selected with.

In addition, we investigated the effect of dose strength, while keeping amoxicillin-clavulanate composition the same (Fig. 5). When we exposed evolved strains to the same amoxicillin proportions at different dose strengths, we found, unsurprisingly, that treatment with higher dose strengths reduced growth to a greater extent (main effect of assay dose strength, β = −0.183, *F*_1,133_ = 338.65, and *P* < 0.0001). We also found that strains that evolved at higher dose strengths were better able to grow in the presence of co-amoxiclav (main effect of selection dose strength, β = 0.116, *F*_1,41_ = 36.66, and *P* < 0.0001) and were less affected when exposed to increased drug doses (selection dose strength by assay dose strength interaction, β = 0.050, χ^2^_1_ = 28.7, and *P* < 0.001).

We found (as before, Fig. 4) that the differences between lines selected at different amoxicillin proportions were stronger for strains selected at high dose strengths (selective dose strength by amoxicillin proportion interaction, β = 0.062, χ^2^_1_ = 11.64, and *P* < 0.001). However, lines did not respond significantly differently to changes in dose strength of co-amoxiclav at different amoxicillin proportions (amoxicillin proportion by assay dose strength interaction, χ^2^_1_ = 0.065 and *P* > 0.5).

In summary, we found that lines selected at higher dose strengths were better adapted to the environment they evolved in, and they were also less affected by changes in dose strengths.

### Genetic changes during selection.

**FIG 6:**
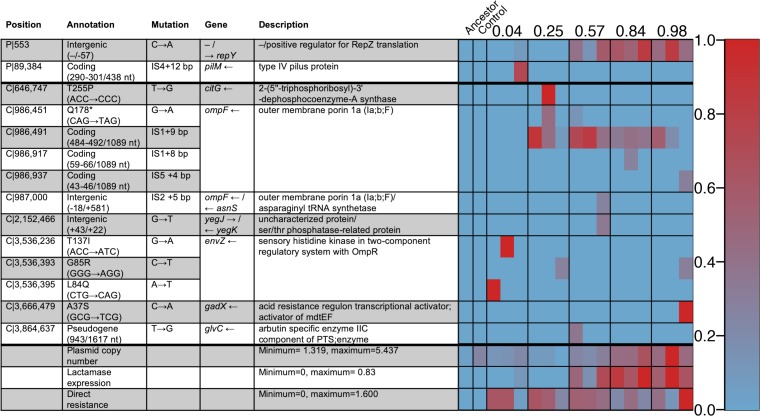
Table and heatmap showing the genetic changes observed in populations adapted to high dose strengths at different amoxicillin/clavulanate proportions. (Left) The columns of the heatmap show the 17 different sequenced populations, with three replicates for the different amoxicillin proportions (listed above). The rows show either genetic changes (found in at least one strain at a frequency of 20% or greater) with the plasmid (P) or chromosomal (C) location or general features of the genomes such as plasmid copy number and summed frequency of mutations giving direct and β-lactamase-mediated resistance to co-amoxicillin. nt, nucleotide; PTS, phosphotransferase system. (Right) Frequencies of individual mutations range from 0% (blue) to 100% (red), while general features range from the minimum and maximum values listed, on the same color scale.

To cast light on the mechanisms of evolved resistance, we sequenced the 15 populations evolved against high dose strengths of co-amoxiclav as well as the ancestral strain and one of the evolved controls. Consistent with our expectation of increased β-lactamase production in low-clavulanate environments (Fig. 2b), we find a pattern of parallel mutation of the plasmid copy number repression locus *repY*, with higher frequency of *repY* mutants in the lines evolved against high-amoxicillin proportions (Fig. 6, *β* = 0.20, *F*_1,13_ = 17.8, and *P* < 0.01). By using read depth of the plasmid and genome regions to estimate plasmid copy number, we find that lines selected against higher amoxicillin proportions indeed evolved higher plasmid copy number (Fig. 6, *β* = 0.20, *F*_1,13_ = 8.46, and *P* < 0.05), and that copy number and *repY* mutations were highly correlated (τ = 0.74 and *P* < 0.0001). Mutations in plasmid copy number control mechanisms are a canonical mechanism to increase plasmid copy number and therefore gene dosing of plasmid cargo—in this case, increased dosing of β-lactamase production (25, 31, 32).

Our model also predicts that mutations conferring β-lactamase-independent resistance will arise independently of the amoxicillin-clavulanate composition. Consistent with this prediction, mutations affecting porins and efflux pumps, which prevent access of amoxicillin to the cell wall target, are also found in multiple lines (Fig. 6), but the frequency of these mutations did not show a significant relationship with the amoxicillin proportion lines were selected against (*F*_1,13_ = 0.0052 and *P* > 0.5).

To further investigate how these two mutation types affect resistance, we tested the 17 sequenced strains against various amoxicillin concentrations again, assaying at more amoxicillin concentrations and with replicate measurements of each replicate selection line (Fig. S5). We find that the frequency of direct resistance mutations in the population increases the growth at all amoxicillin proportions (main effect of direct resistance, β = 0.042, χ^2^_1_ = 4.19, and *P* < 0.05), but the effect did not vary with amoxicillin proportion (direct resistance by assay amoxicillin proportion interaction, χ^2^_1_ = 0.778 and *P* > 0.1). On the other hand, mutations in *repY* had a significant interaction with amoxicillin proportion so that these populations grew better at high amoxicillin proportions (*repY* mutation frequency by amoxicillin proportion interaction, β = 0.024, χ^2^_1_ = 5.83, and *P* < 0.05). This further suggests that mutations that increased β-lactamase expression are fitter when the ratio of amoxicillin to clavulanate is high.

10.1128/mBio.01831-19.5 FIG S5 The growth of evolved strains at different amoxicillin proportions is a function of their direct and β-lactamase-mediated resistance. (a) The resistance profile of the sequenced strains in terms of mutations that change their β-lactamase expression (*repY* mutations) and mutations that directly resist amoxicillin (mutations in genes encoding porins and efflux pumps). The points are labeled according to the strains they represent so that the first number represents the selection amoxicillin proportion (or Anc and EC for ancestor and evolutionary control, respectively), and the second number represents the replicate. (b) Growth of strains after 22 hours at different amoxicillin proportions (at high dose strength). Direct resistance increases growth by the same amount at all amoxicillin proportions, whereas β-lactamase-mediated resistance positively affects growth at all amoxicillin proportions but has a stronger effect at high amoxicillin proportions. Points are the mean of three replicates. Lines are fits from a mixed-effects linear model described in the main article. For amoxicillin proportions of 0.10 and 0.18, the concentrations of amoxicillin were 1.31 and 1.75 μg/ml, respectively, paired with clavulanate concentrations of 15.7 and 10.3 μg/ml. Download FIG S5, TIF file, 0.2 MB. Copyright © 2019 Allen and Brown. 2019 Allen and Brown This content is distributed under the terms of the Creative Commons Attribution 4.0 International license.

## DISCUSSION

In this study, we have demonstrated that the synergistic interaction between a β-lactam antibiotic and a β-lactamase inhibitor (adjuvant) can lead to distinct phenotypic and genomic paths to resistance evolution in a ratio-dependent manner, with potential consequences for the sustainable management of adjuvant therapies. Several studies have demonstrated that the ratio of drugs used in combination therapies can affect selection for resistance (30, 33, 34). However, the conclusions of these studies are often in terms of how the ratio of the different drug components affects the strength of inhibition, which has a well-established effect on the evolution of drug resistance (3). We find that low-amoxicillin/high-clavulanate treatments confer weaker selection for resistance (Fig. 3b), even when the inhibitory effect of the drug combination on the ancestor (Fig. 3a) and populations evolved in the absence of antibiotics (Fig. 3c) is not dependent on the drug ratio.

Our drug concentrations were chosen so that inhibition, measured by final optical density, was not affected by amoxicillin proportion (Fig. 3). However, the low-amoxicillin-proportion treatments affect growth more when we measure CFU (see Fig. S1 and S2 in the supplemental material). Stronger inhibition at low-amoxicillin proportions cannot explain the differences we find in rates of adaptation, as we (and others [3, 30]) show that resistance generally evolves faster when the inhibitory effect is greater (Fig. 3 and 5). In contrast, we found that all lines selected in low-amoxicillin/high-clavulanate environments had the least resistance, regardless of whether they were evolved at high or low dose strengths (Fig. 3d).

Our mathematical model (Fig. 2b) suggests that increased β-lactamase expression is more strongly selected with high proportions of amoxicillin (relative to clavulanate), because when clavulanate is not in excess, its effect can simply be titrated out by increasing β-lactamase expression. On the other hand, selection for β-lactamase-independent resistance depends only on the inhibitory strength of the drug combination, because this is equivalent to the amoxicillin concentration experienced by the bacterium after some proportion has been broken down by β-lactamase. Consistent with our model, we found that lines selected at high amoxicillin proportions grew well in high-amoxicillin environments but poorly in low-amoxicillin/high-clavulanate environments (Fig. 4c to e). These lines had increased plasmid copy number (and thus β-lactamase expression), likely due to mutations in *repY* (Fig. 6). Increased plasmid copy number, and thus increased β-lactamase expression, protects against amoxicillin but less so in the presence of high levels of clavulanate (Fig. S5). On the other hand, lines selected in high-clavulanate environments grew poorly but consistently across all amoxicillin proportions (Fig. 4a). These lines acquired direct resistance to amoxicillin only through parallel mutations affecting porins and efflux pumps, a resistance mechanism seen across all amoxicillin-clavulanate compositions (Fig. 6). This resistance mechanism provides a benefit independent of amoxicillin proportion, as it depends only on the amount of noncleaved amoxicillin (Fig. 2a and Fig. S5).

In addition to mutations in existing genetic material, clinical strains can also acquire resistance genes via horizontal gene transfer (HGT)—including acquisition of β-lactamases (35, 36). Our genetically closed experimental model system precludes the HGT acquisition of novel resistance genes; however, our results provide a framework to make predictions on the role of HGT in the context of co-amoxiclav selection. We expect the logic of clavulanate titration to hold regardless of whether changes in β-lactamase activity are driven by single nucleotide polymorphisms (SNPs) or HGT, with greater clavulanate concentrations providing a stronger barrier to β-lactamase mutant selection. However, we expect differences in the dose response. For HGT-acquired β-lactamase enzymes that are more effective at amoxicillin cleavage and/or more resistant to clavulanate inhibition, we expect a shallower clavulanate dose-response curve and therefore predict that more clavulanate would be required to inhibit β-lactamase enrichment under co-amoxiclav selection.

Although the shapes of drug interactions have recently been shown to evolve in bacteria (34), to our knowledge, this is the first time that this has been reported for antibiotic-adjuvant combinations or that these changes have been linked to the mechanisms of drug action. Our results suggest that dosing regimens with larger amounts of clavulanate will more effectively slow the evolution of resistance by rendering some resistance types ineffective; specifically, β-lactamase dose-response mutations will be less able to titrate out the effect of larger amounts of β-lactamase inhibitor. There are a number of caveats when relating our *in vitro* experimental evolution work to a clinical context. First, in a clinical context, drug doses are designed to exceed MIC values (at least transiently) and will vary through time and across tissue location. Our experimental evolution approach takes a vastly simplifying approach to this complexity by focusing only on the window of sub-MICs that are likely to drive resistance evolution. Second, the meaning of “treatment efficacy” changes in a clinical context, where we have access to (far more important) metrics of patient health. In our experimental evolution microcosm, there is no host to measure, so we must rely on simple proxies of bacterial growth. Overall, we view our approach as a first step toward animal model studies and ultimately human clinical trials of altered antibiotic-adjuvant formulations.

Since its introduction, the dosage of amoxicillin in co-amoxiclav tablets has increased from 250 mg to 875 mg to combat amoxicillin resistance; however, the dosage of clavulanate has remained the same at 125 mg (24). There are many other considerations when designing dosing regimens including pharmacokinetics/pharmacodynamics and toxicity (although amoxicillin and clavulanate are well tolerated [37, 38]), but the potential for resistance is increasingly recognized as an important consideration in treatment design. Increased β-lactamase expression is a common resistance mechanism, particularly when plasmid borne (25). Therefore, we suggest that increased clavulanate dosing should be considered in a clinical context as a method to reduce selection for increased β-lactamase expression without affecting the fitness of other resistance mechanisms. This would have the added benefit of reducing selection for plasmid-based resistance, which is both easily mobilized and can increase evolvability (25).

## MATERIALS AND METHODS

### Strains and media.

Escherichia coli strain MG1655 transformed with a pCT plasmid and defective for horizontal transfer due to a mutation in the *trbA* gene (39) was used as the ancestor of all selection lines and is referred to as the ancestor in the text. The strain was produced in the lab of Ben Raymond (39) and kindly provided. The pCT plasmid is a large naturally occurring plasmid containing the CTX-M-14 extended-spectrum β-lactamase. The pCT plasmid is stable; however, prior to incubation for experimental evolution and growth dynamics assays, the ancestor was grown in the presence of 100 μg/ml ampicillin to maintain the pCT plasmid. For phenotyping of experimentally evolved strains, preculture was performed without antibiotics to reduce any nongenetic effects of exposure to antibiotic treatment.

All assays were conducted in a defined minimal medium. The defined minimal medium consisted of M9 medium base (containing 6.78 mg/ml Na_2_HPO_4_, 3 mg/ml KH_2_PO_4_, 0.5 mg/ml NaCl, and 1 mg/ml NH_4_Cl) supplemented with 1 mM MgSO_4_, 0.1 mM CaCl_2_, 0.4% (vol/vol) glycerol, 0.02% Casamino Acids, 0.5 μg/ml thiamine, and Hutners trace elements (40).

Clavulanate (in the form of potassium clavulanate; Fluka Analytical) and amoxicillin (LKT Laboratories) were supplied in powdered forms, stored at 4°C, and used to make stocks in deionized water. These stocks were stored at 4°C according to the manufacturer’s instructions, and liquid stocks were not kept for longer than 14 days to minimize degradation of the compounds.

To test antibiotic sensitivity of the ancestral strain, the ancestor was grown for 22 h in M9 in the presence of increasing clavulanate and amoxicillin, at all possible combinations of the two drug concentrations (checkerboard assay in Fig. 1b; see also Fig. S1 in the supplemental material). From these, five different ratios of amoxicillin and clavulanate as well as associated iso-inhibitory doses (absolute concentrations of both amoxicillin and clavulanate that inhibited final yield after 22 h to a similar extent) were identified for each ratio. The chosen concentrations are shown in Table 1 and plotted relative to the checkerboard plot in Fig. 1b. CFU counting of diluted cultures (after 22-h growth) spread on antibiotic-free LB agar plates was used as an alternate metric of growth.

### Experimental evolution.

To test its ability to adapt to different drug doses, the ancestor was selected against various drug regimens defined by the relative proportion of amoxicillin and dose strength (Table 1, Fig. 1b, and Fig. S3a). A mid-exponential culture of the ancestor was washed and diluted in minimal medium. This was aliquoted into 48 wells in the center of a 96-well plate, which were then made up to a final volume of 200 μl by adding reconstituted clavulanate and amoxicillin, so that starting densities were optical densities at 600 nm (OD_600_) of 0.01. Experimental evolution lines were set up corresponding to five drug ratios at three different dose strengths, plus one line which was not exposed to drugs, each replicated three times (48 independent lines), plus three replicate sterile wells with no drugs (which were still passaged).

The plates were incubated statically at 37°C for 22 h for each passage (to allow time for passaging within a day). After each growth cycle, wells were mixed using a pipette to resuspend any clumps of bacteria. The optical density of the wells was then measured and used to transfer cells to a fresh microwell plate so that each line started at an OD_600_ of 0.01. Experimental evolution was performed for 12 passages (corresponding to approximately 84 generations). Lines were frozen every three passages by adding 100 μl of a 1:1 LB-glycerol mixture to the remaining culture after the transfer had been performed; these were then frozen at –80°C.

### Measuring cross-resistance between drug environments.

For each selection line, the population after six passages (chosen because this is when there was the most diversity in how lines had adapted to their environment) was revived by overnight growth in LB. Each line of selection was assayed for growth at the dose strength and amoxicillin proportion it was selected at and in drug environments with either altered amoxicillin proportion or altered dose strength. When varying dose strength, an increased dose of 1.15 times the maximum dose was also used. Otherwise, all conditions were the same, strains were grown in minimal media statically for 22 h at 37°C and mixed prior to measuring optical density; again CFU counting was used as an additional growth measure for some conditions.

As these experiments did not fit on a single 96-well plate, conditions were randomly blocked across several 96-well plates. Where there was significant (yet small) variation in the growth of control lines across plates, OD values for each plate were corrected using the growth of controls (in the absence of drugs). Control lines (ancestral strain and evolved controls) were exposed to all conditions.

### Statistics.

All statistics were performed in R (41). The parameters of the theoretical model were estimated by fitting equation 1 to the checkerboard data using a nonlinear least-squares estimation (nls). This analysis showed that the exponents *x* and *y* were not significantly different, so these were represented by a single parameter. The surface in Fig. 1b is a loess smoothed fit so as to independently show the pattern of synergy.

To model the final density of evolved strains grown in different drug environments, we used linear mixed-effects models (nlme package [42]) with selection line used as a random effect (on intercept). The fixed effects were selection dose strength, selection amoxicillin proportion, and either assay amoxicillin proportion (Fig. 4) or assay dose strength (Fig. 5), as well as their two term interactions. Dose strengths and amoxicillin proportions were encoded as continuous parameters to reflect their continuous nature and order between them (Table 1); these models did not include drug-free treatments as amoxicillin proportion is undefined. Dose strength was 0, 1, or 2 for the low to high doses (with increased dose coded as 3 where appropriate). Note that zero for dose strength represents the lowest dose (not the absence of drug). Amoxicillin proportions were input as the log of amoxicillin proportion, to reflect the strong effect of low amoxicillin proportions. CFU data were square root transformed to fit model assumptions. Nonsignificant terms (based on likelihood ratio [χ^2^] tests and F tests for mixed-effects and fixed-effects models, respectively) were dropped to simplify models. Similar models were used for the effect of different types of resistance mutations, but the frequencies of direct or β-lactamase-mediated resistance were used as predictors in place of selection conditions.

To fit the growth of the selection lines over the course of the selection experiment, we fitted a Gompertz equation (43) to describe the OD as a function of number of passages, using a nonlinear mixed-effects model (nlme package [42]).

(4) $$\text{OD}∼Ae^{-c\;{\left(1-k\right)}^{\text{Passage}}}$$

where *A* controls the maximum value, *c* controls the intercept, and *k* controls how quickly the function increases. We allowed the three parameters of the Gompertz fit to vary according to the two parameters of selection environment (amoxicillin proportion and dose strength). Selection line was included as a random effect influencing intercept (*c*). We then used likelihood ratio tests to test for significant effects of the two main effects and the interaction on each parameter, dropping nonsignificant effects (not involved in higher-order interactions) to simplify the model.

### Sequencing and bioinformatics.

To test whether different drug ratios select for different resistance mutations, we sequenced evolved populations selected against the highest dose strengths after six passages of experimental evolution. The ancestral strain and one of the three populations that evolved in the absence of drugs were also sequenced. Library preparation and paired-end MiSeq sequencing was performed by Edinburgh Genomics. Obtained sequences were aligned to both the E. coli MG1655 reference (44) and the pCT plasmid reference (45), and polymorphisms were identified using breseq in polymorphism mode using default parameters (46, 47). These default breseq settings identified a stable polymorphism in all samples (including ancestor) in a repeat region with high coverage—indicating a misaligned repeat. Manual resolution of this region (introducing an additional repeat region into the ancestor genome) resulted in successful mapping to a single ancestor genome that was used to call mutations. Plasmid copy number was calculated as the ratio of average plasmid read depth to average chromosome read depth. Mutations considered as affecting plasmid copy number (*repY*) and direct resistance (efflux pumps and porins) were identified from genome annotation and are marked in the mutations table of the Dryad entry.

### Data availability.

Single nucleotide polymorphism (SNP) tables relative to the E. coli MG1655 reference for resequencing data and raw data from experiments are available at Dryad (https://doi.org/10.5061/dryad.fr8pm0n).
