# Supplementary figures and images for: Modified Antibiotic Adjuvant Ratios Can Slow and Steer the Evolution of Resistance: Co-amoxiclav as a Case Study
Source: mBio. 2019 Sep 17;10(5):e01831-19. doi: 10.1128/mBio.01831-19 (PMC6751059; doi:10.1128/mBio.01831-19)

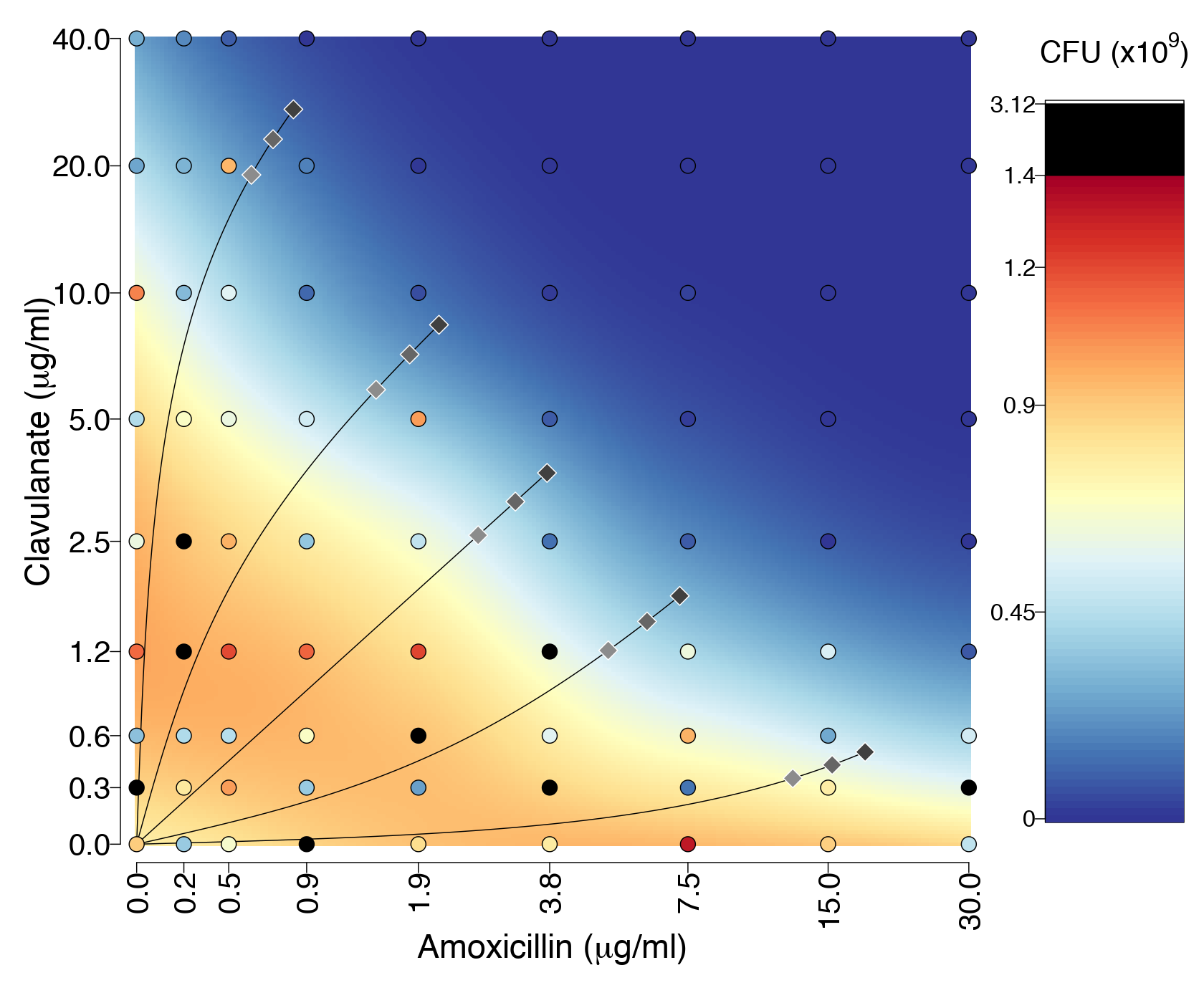

Supplement: FIG S1 [file mBio.01831-19-sf001.tif]

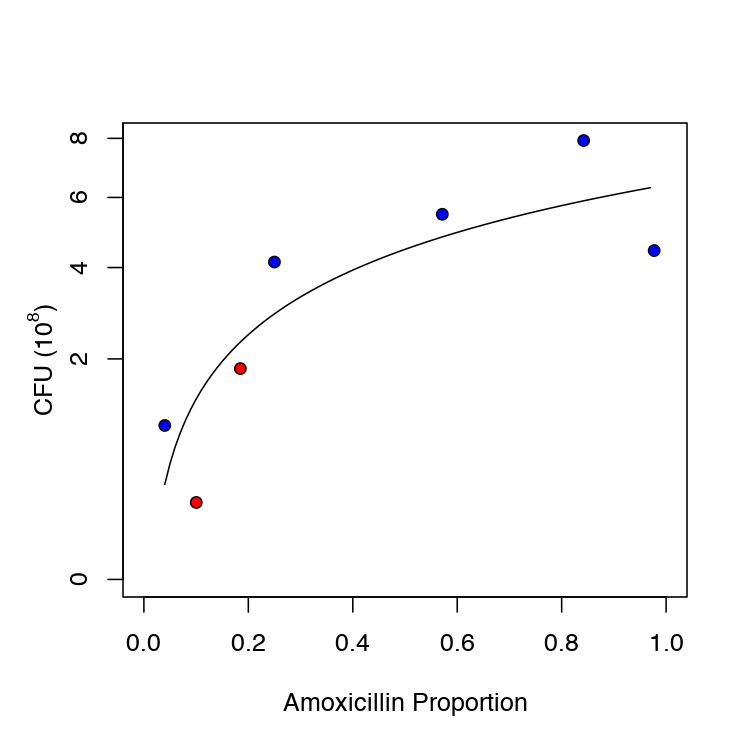

Supplement: FIG S2 [file mBio.01831-19-sf002.tif]

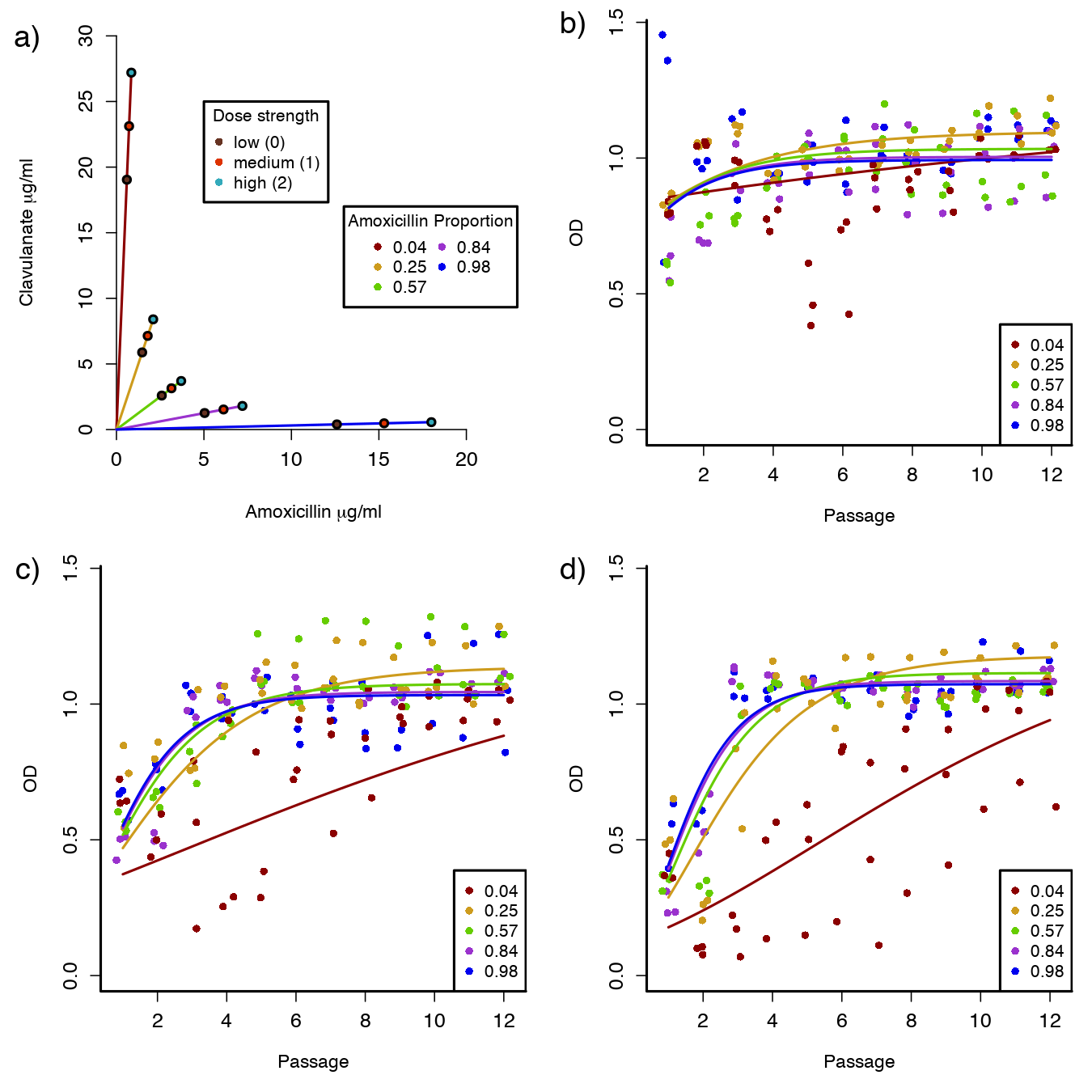

Supplement: FIG S3 [file mBio.01831-19-sf003.tif]

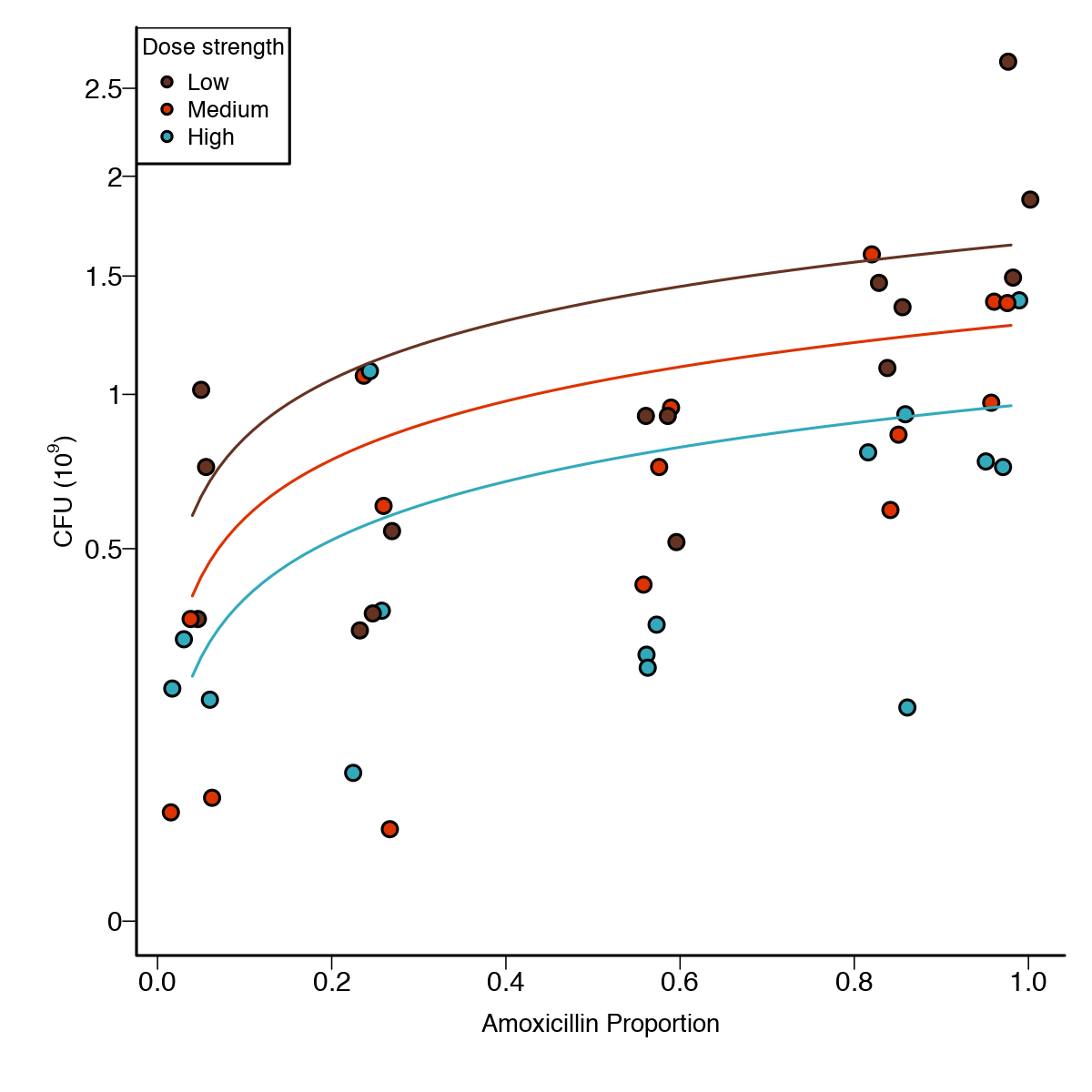

Supplement: FIG S4 [file mBio.01831-19-sf004.tif]

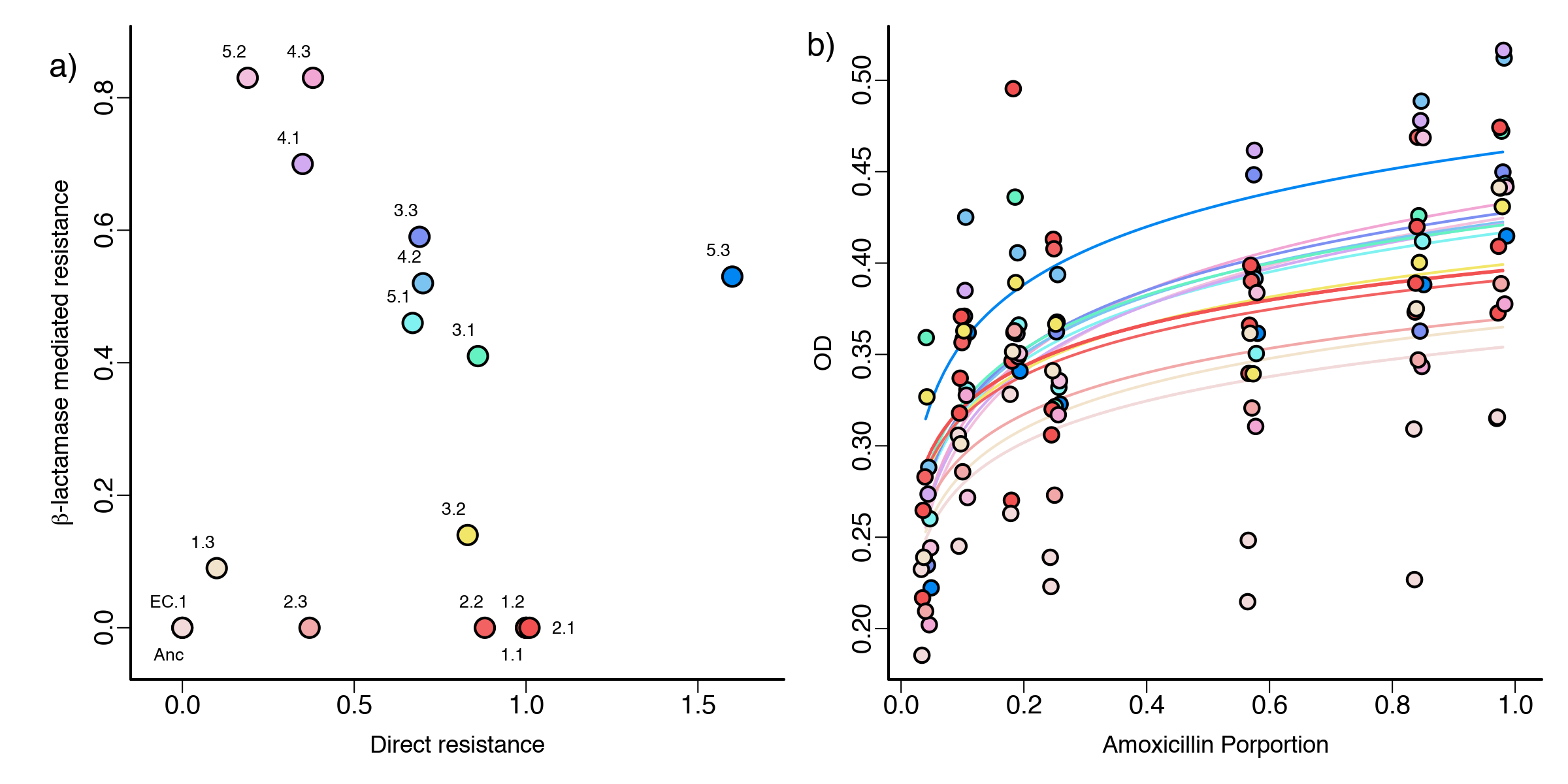

Supplement: FIG S5 [file mBio.01831-19-sf005.tif]
